# A New Method of Producing Local Anæsthesia

**Published:** 1890-11

**Authors:** 


					﻿A New Method of Producing Local Anesthesia.—Dr. Voit-
uriez recommends in the Moniteur Therapeutique a method of
producing local anaesthesia which certainly possesses the
merit of simplicity. It is based upon the “ well-known anaes-
thetic properties of carbonic oxide,” and consists in pouring
on the place to be anaesthetized the contents of two or three
bottles of Seltzer water, preferably by means of the siphon,
which releases the water in a strong stream. The anaesthesia
lasts five minutes and then gradually disappears. It is diffi-
cult to see how the “well-known anaesthetic properties” of the
oxide are exerted by external application. The effect, if any,
is probably mechanical.—Drugists' Circular and Chemical Gaz.
				

